# 8-Hy­droxy-2-methyl­quinolinium tetra­chlorido(pyridine-2-carboxyl­ato-κ^2^ *N*,*O*)stannate(IV)

**DOI:** 10.1107/S1600536811001942

**Published:** 2011-01-22

**Authors:** Ezzatollah Najafi, Mostafa M. Amini, Seik Weng Ng

**Affiliations:** a Department of Chemistry, General Campus, Shahid Beheshti University, Tehran 1983963113, Iran; b Department of Chemistry, University of Malaya, 50603 Kuala Lumpur, Malaysia

## Abstract

In the reaction of pyridine-2-carb­oxy­lic acid and stannic chloride in the presence of 2-methyl-8-hy­droxy­quinoline, the 2-methyl-8-hy­droxy­quinoline is protonated, yielding the title salt, (C_10_H_10_NO)[SnCl_4_(C_6_H_4_NO_2_)]. The Sn^IV^ atom in the anion is *N*,*O*-chelated by a pyridine-2-carboxyl­ate in a *cis*-SnNOCl_4_ octa­hedral geometry. The cation is linked to the anion by an O—H⋯O hydrogen bond.

## Related literature

For other 8-hy­droxy-2-methyl­quinolinium salts, see: Najafi *et al.* (2010 ▶); Sattarzadeh *et al.* (2009 ▶).
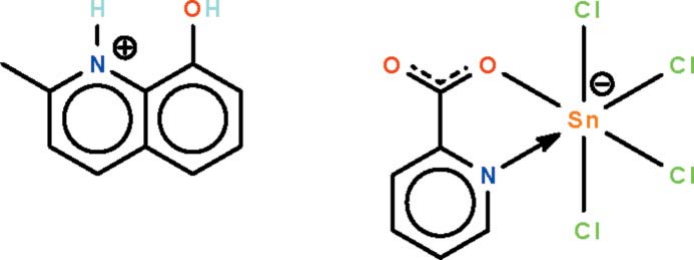


## Experimental

### 

#### Crystal data

(C_10_H_10_NO)[SnCl_4_(C_6_H_4_NO_2_)]
*M* *_r_* = 542.78
Monoclinic, 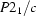

*a* = 11.5188 (2) Å
*b* = 11.1971 (2) Å
*c* = 15.0257 (2) Å
β = 94.563 (2)°
*V* = 1931.83 (5) Å^3^
*Z* = 4
Mo *K*α radiation
μ = 1.90 mm^−1^
*T* = 100 K
0.30 × 0.25 × 0.20 mm

#### Data collection

Agilent SuperNova Dual diffractometer with an Atlas detector
Absorption correction: multi-scan (*CrysAlis PRO*; Agilent Technologies, 2010 ▶) *T*_min_ = 0.600, *T*_max_ = 0.703
9737 measured reflections
4304 independent reflections
3686 reflections with *I* > 2σ(*I*)
*R*_int_ = 0.031

#### Refinement

*R*[*F* ^2^ > 2σ(*F* ^2^)] = 0.028
*wR*(*F* ^2^) = 0.064
*S* = 1.04
4304 reflections
242 parameters
2 restraints
H atoms treated by a mixture of independent and constrained refinement
Δρ_max_ = 0.54 e Å^−3^
Δρ_min_ = −0.62 e Å^−3^

### 

Data collection: *CrysAlis PRO* (Agilent Technologies, 2010 ▶); cell refinement: *CrysAlis PRO*; data reduction: *CrysAlis PRO*; program(s) used to solve structure: *SHELXS97* (Sheldrick, 2008 ▶); program(s) used to refine structure: *SHELXL97* (Sheldrick, 2008 ▶); molecular graphics: *X-SEED* (Barbour, 2001 ▶); software used to prepare material for publication: *publCIF* (Westrip, 2010 ▶).

## Supplementary Material

Crystal structure: contains datablocks global, I. DOI: 10.1107/S1600536811001942/si2327sup1.cif

Structure factors: contains datablocks I. DOI: 10.1107/S1600536811001942/si2327Isup2.hkl

Additional supplementary materials:  crystallographic information; 3D view; checkCIF report

## Figures and Tables

**Table 1:** Hydrogen-bond geometry (Å, °)

*D*—H⋯*A*	*D*—H	H⋯*A*	*D*⋯*A*	*D*—H⋯*A*
O3—H3⋯O2	0.84 (3)	1.86 (1)	2.686 (3)	168 (3)

**Fig. 1.**
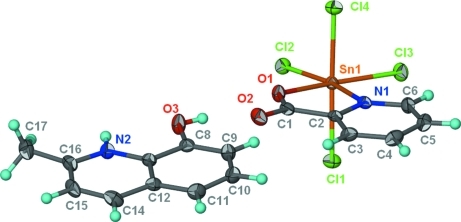
Thermal ellipsoid plot (Barbour, 2001) of [C10H10NO2]+ [SnCl4(C6H4NO2)]– at the 70% probability level; hydrogen atoms are drawn as spheres of arbitrary radius.

## supplementary crystallographic information

### Comment

The direct synthesis of a potentially chelating amino-carboxylic acid with stannic tetrachloride has not been reported. Pyridine-2-carboxylic acid yields a number of derivatives with organotin compounds; these are either synthesized by condensing the amino-carboxylic acids with an organotin oxide/hydroxide or by reacting the amino-carboxylic acids with an organotin chloride in the presence of a proton abstractor. With the latter route, the product may be an organostannate in which the pyridine-2-carboxylate chelates to the chlorine-bonded tin atom. In the reaction of pyridine-2-carboxylic acid and stannic chloride in the presence of 2-methyl-8-hydroxyquinoline, the 2-methyl-8-hydroxyquinoline is protonated to yield the salt, [C_10_H_10_NO_2_]^+^ [SnCl_4_(C_6_H_4_NO_2_)]^-^ (Scheme I, Fig. 1). The tin atom in the anion is *N*,*O*-chelated by a pyridine-2-carboxylate in an octahedral geometry. The cation is linked to the anion by an O–H···O hydrogen bond (Table 1). The cation been observed in a similar reaction with zinc salts (Najafi *et al.*, 2010; Sattarzadeh *et al.*, 2009).

### Experimental

Stannic chloride pentahydrate (0.35 g, 1 mmol), pyridine-2-carboxylic acid (0.13 g, 1 mmol) and 2-methyl-8-hydroxyquinoline (0.16 g, 1 mmol) were loaded into a convection tube; the tube was filled with dry methanol and kept at 333 K. Beige crystals were collected from the side arm after several days.

### Refinement

Carbon-bound H-atoms were placed in calculated positions [C—H 0.95 to 0.98 Å, *U*_iso_(H) 1.2 to 1.5*U*_eq_(C)] and were included in the refinement in the riding model approximation.

The amino and hydroxy H-atoms were located in a difference Fourier map, and were refined with distance restraints of N–H 0.88±0.01, O–H 0.84±0.01 Å; their temperature factors were refined.

### Crystal data

**Table:**

(C_10_H_10_NO)[SnCl_4_(C_6_H_4_NO_2_)]	*F*(000) = 1064
*M**_r_* = 542.78	*D*_x_ = 1.866 Mg m^−^^3^
Monoclinic, *P*2_1_/*c*	Mo *K*α radiation, λ = 0.71073 Å
Hall symbol: -P 2ybc	Cell parameters from 5318 reflections
*a* = 11.5188 (2) Å	θ = 2.3–29.2°
*b* = 11.1971 (2) Å	µ = 1.90 mm^−^^1^
*c* = 15.0257 (2) Å	*T* = 100 K
β = 94.563 (2)°	Prism, beige
*V* = 1931.83 (5) Å^3^	0.30 × 0.25 × 0.20 mm
*Z* = 4	

### Data collection

**Table:**

Agilent SuperNova Dual diffractometer with an Atlas detector	4304 independent reflections
Radiation source: SuperNova (Mo) X-ray Source	3686 reflections with *I* > 2σ(*I*)
Mirror	*R*_int_ = 0.031
Detector resolution: 10.4041 pixels mm^-1^	θ_max_ = 27.5°, θ_min_ = 2.5°
ω scans	*h* = −13→14
Absorption correction: multi-scan (*CrysAlis PRO*; Agilent Technologies, 2010)	*k* = −9→14
*T*_min_ = 0.600, *T*_max_ = 0.703	*l* = −19→18
9737 measured reflections	

### Refinement

**Table:**

Refinement on *F*^2^	Primary atom site location: structure-invariant direct methods
Least-squares matrix: full	Secondary atom site location: difference Fourier map
*R*[*F*^2^ > 2σ(*F*^2^)] = 0.028	Hydrogen site location: inferred from neighbouring sites
*wR*(*F*^2^) = 0.064	H atoms treated by a mixture of independent and constrained refinement
*S* = 1.04	*w* = 1/[σ^2^(*F*_o_^2^) + (0.0264*P*)^2^] where *P* = (*F*_o_^2^ + 2*F*_c_^2^)/3
4304 reflections	(Δ/σ)_max_ = 0.001
242 parameters	Δρ_max_ = 0.54 e Å^−^^3^
2 restraints	Δρ_min_ = −0.62 e Å^−^^3^

### Fractional atomic coordinates and isotropic or equivalent isotropic displacement parameters (Å^2^)

**Table:**

	*x*	*y*	*z*	*U*_iso_*/*U*_eq_	
Sn1	0.739358 (15)	0.452510 (16)	0.285941 (11)	0.01301 (7)	
Cl1	0.63844 (6)	0.27357 (6)	0.32327 (4)	0.01928 (15)	
Cl2	0.57101 (6)	0.54218 (6)	0.21479 (4)	0.01787 (15)	
Cl3	0.81600 (6)	0.36052 (6)	0.15995 (4)	0.02070 (16)	
Cl4	0.85945 (6)	0.62635 (6)	0.26734 (4)	0.01903 (15)	
O1	0.70258 (16)	0.52449 (16)	0.41026 (12)	0.0163 (4)	
O2	0.75372 (17)	0.52641 (17)	0.55626 (12)	0.0202 (4)	
O3	0.66210 (17)	0.64322 (18)	0.69025 (12)	0.0206 (4)	
H3	0.690 (3)	0.616 (3)	0.6447 (13)	0.031*	
N1	0.88239 (18)	0.38362 (19)	0.38018 (13)	0.0131 (5)	
N2	0.58299 (19)	0.7069 (2)	0.84655 (14)	0.0154 (5)	
H2	0.553 (2)	0.732 (3)	0.7947 (11)	0.023*	
C1	0.7667 (2)	0.4919 (2)	0.48003 (17)	0.0153 (6)	
C2	0.8656 (2)	0.4085 (2)	0.46593 (17)	0.0138 (5)	
C3	0.9373 (2)	0.3617 (2)	0.53524 (18)	0.0180 (6)	
H3A	0.9239	0.3787	0.5955	0.022*	
C4	1.0291 (2)	0.2895 (2)	0.51533 (18)	0.0196 (6)	
H4	1.0788	0.2552	0.5619	0.024*	
C5	1.0481 (2)	0.2677 (3)	0.42687 (18)	0.0191 (6)	
H5	1.1125	0.2207	0.4119	0.023*	
C6	0.9718 (2)	0.3155 (2)	0.36087 (18)	0.0174 (6)	
H6	0.9832	0.2994	0.3001	0.021*	
C8	0.7199 (2)	0.5982 (2)	0.76441 (17)	0.0155 (6)	
C9	0.8168 (2)	0.5276 (2)	0.76669 (18)	0.0194 (6)	
H9	0.8488	0.5065	0.7125	0.023*	
C10	0.8698 (2)	0.4858 (3)	0.84826 (19)	0.0205 (6)	
H10	0.9385	0.4388	0.8483	0.025*	
C11	0.8247 (2)	0.5114 (3)	0.92770 (18)	0.0203 (6)	
H11	0.8598	0.4796	0.9820	0.024*	
C12	0.7261 (2)	0.5851 (2)	0.92813 (18)	0.0161 (6)	
C13	0.6761 (2)	0.6306 (2)	0.84629 (17)	0.0149 (6)	
C14	0.6737 (2)	0.6196 (3)	1.00615 (18)	0.0211 (6)	
H14	0.7031	0.5884	1.0623	0.025*	
C15	0.5820 (2)	0.6965 (3)	1.00250 (18)	0.0203 (6)	
H15	0.5489	0.7192	1.0559	0.024*	
C16	0.5358 (2)	0.7424 (3)	0.91984 (18)	0.0187 (6)	
C17	0.4394 (3)	0.8305 (3)	0.91255 (19)	0.0237 (7)	
H17A	0.4068	0.8350	0.8504	0.036*	
H17B	0.4692	0.9091	0.9318	0.036*	
H17C	0.3786	0.8056	0.9506	0.036*	

### Atomic displacement parameters (Å^2^)

**Table:**

	*U*^11^	*U*^22^	*U*^33^	*U*^12^	*U*^13^	*U*^23^
Sn1	0.01389 (11)	0.01420 (11)	0.01113 (10)	0.00037 (7)	0.00216 (7)	−0.00015 (7)
Cl1	0.0187 (3)	0.0184 (3)	0.0206 (3)	−0.0032 (3)	0.0005 (3)	0.0025 (3)
Cl2	0.0166 (3)	0.0192 (3)	0.0174 (3)	0.0018 (3)	−0.0008 (3)	0.0015 (3)
Cl3	0.0250 (4)	0.0234 (4)	0.0142 (3)	0.0023 (3)	0.0047 (3)	−0.0034 (3)
Cl4	0.0202 (3)	0.0173 (3)	0.0198 (3)	−0.0032 (3)	0.0028 (3)	0.0020 (3)
O1	0.0187 (10)	0.0195 (10)	0.0107 (9)	0.0043 (8)	0.0020 (8)	−0.0013 (8)
O2	0.0233 (11)	0.0247 (11)	0.0132 (10)	−0.0022 (9)	0.0048 (8)	−0.0031 (8)
O3	0.0230 (11)	0.0263 (11)	0.0125 (10)	0.0091 (9)	0.0014 (8)	−0.0013 (8)
N1	0.0141 (11)	0.0139 (11)	0.0115 (11)	−0.0029 (10)	0.0018 (9)	−0.0009 (9)
N2	0.0163 (12)	0.0177 (12)	0.0121 (11)	0.0015 (10)	0.0001 (9)	−0.0005 (10)
C1	0.0180 (14)	0.0147 (14)	0.0138 (14)	−0.0040 (12)	0.0048 (11)	−0.0017 (11)
C2	0.0145 (13)	0.0124 (13)	0.0148 (13)	−0.0044 (11)	0.0024 (10)	0.0004 (11)
C3	0.0237 (15)	0.0145 (14)	0.0158 (14)	−0.0030 (12)	0.0020 (12)	−0.0004 (11)
C4	0.0225 (15)	0.0165 (14)	0.0190 (14)	0.0006 (12)	−0.0044 (12)	0.0017 (12)
C5	0.0164 (14)	0.0181 (14)	0.0224 (15)	0.0020 (12)	−0.0004 (11)	−0.0031 (12)
C6	0.0153 (14)	0.0170 (14)	0.0204 (15)	0.0007 (12)	0.0038 (11)	−0.0026 (12)
C8	0.0171 (14)	0.0137 (13)	0.0159 (13)	−0.0030 (12)	0.0017 (11)	−0.0011 (11)
C9	0.0227 (15)	0.0192 (15)	0.0170 (14)	0.0009 (12)	0.0058 (12)	−0.0034 (12)
C10	0.0165 (14)	0.0184 (14)	0.0269 (16)	0.0027 (12)	0.0030 (12)	0.0015 (12)
C11	0.0220 (15)	0.0211 (15)	0.0172 (15)	0.0015 (13)	−0.0015 (12)	0.0045 (12)
C12	0.0155 (14)	0.0151 (14)	0.0178 (14)	−0.0030 (12)	0.0014 (11)	0.0010 (11)
C13	0.0152 (14)	0.0144 (14)	0.0152 (13)	−0.0018 (11)	0.0024 (11)	0.0003 (11)
C14	0.0194 (15)	0.0283 (17)	0.0155 (14)	−0.0055 (13)	0.0007 (11)	0.0025 (12)
C15	0.0226 (15)	0.0265 (16)	0.0127 (13)	−0.0029 (13)	0.0079 (11)	−0.0057 (12)
C16	0.0168 (14)	0.0207 (15)	0.0195 (14)	−0.0050 (12)	0.0063 (11)	−0.0049 (12)
C17	0.0224 (16)	0.0277 (17)	0.0219 (15)	0.0018 (13)	0.0064 (12)	−0.0050 (13)

### Geometric parameters (Å, °)

**Table:**

Sn1—O1	2.1081 (17)	C5—C6	1.380 (4)
Sn1—N1	2.223 (2)	C5—H5	0.9500
Sn1—Cl2	2.3626 (7)	C6—H6	0.9500
Sn1—Cl3	2.3854 (7)	C8—C9	1.365 (4)
Sn1—Cl1	2.4050 (7)	C8—C13	1.414 (4)
Sn1—Cl4	2.4171 (7)	C9—C10	1.405 (4)
O1—C1	1.286 (3)	C9—H9	0.9500
O2—C1	1.229 (3)	C10—C11	1.369 (4)
O3—C8	1.350 (3)	C10—H10	0.9500
O3—H3	0.84 (3)	C11—C12	1.404 (4)
N1—C6	1.332 (3)	C11—H11	0.9500
N1—C2	1.347 (3)	C12—C13	1.411 (4)
N2—C16	1.327 (3)	C12—C14	1.414 (4)
N2—C13	1.371 (3)	C14—C15	1.361 (4)
N2—H2	0.88 (3)	C14—H14	0.9500
C1—C2	1.502 (4)	C15—C16	1.409 (4)
C2—C3	1.380 (4)	C15—H15	0.9500
C3—C4	1.382 (4)	C16—C17	1.483 (4)
C3—H3A	0.9500	C17—H17A	0.9800
C4—C5	1.386 (4)	C17—H17B	0.9800
C4—H4	0.9500	C17—H17C	0.9800
			
O1—Sn1—N1	76.07 (7)	C4—C5—H5	120.6
O1—Sn1—Cl2	91.31 (5)	N1—C6—C5	121.7 (3)
N1—Sn1—Cl2	167.32 (6)	N1—C6—H6	119.2
O1—Sn1—Cl3	168.94 (5)	C5—C6—H6	119.2
N1—Sn1—Cl3	93.06 (6)	O3—C8—C9	125.9 (2)
Cl2—Sn1—Cl3	99.60 (2)	O3—C8—C13	115.8 (2)
O1—Sn1—Cl1	88.68 (5)	C9—C8—C13	118.3 (2)
N1—Sn1—Cl1	84.83 (6)	C8—C9—C10	120.8 (3)
Cl2—Sn1—Cl1	93.73 (2)	C8—C9—H9	119.6
Cl3—Sn1—Cl1	92.39 (2)	C10—C9—H9	119.6
O1—Sn1—Cl4	87.23 (5)	C11—C10—C9	121.5 (3)
N1—Sn1—Cl4	87.20 (6)	C11—C10—H10	119.3
Cl2—Sn1—Cl4	93.56 (2)	C9—C10—H10	119.3
Cl3—Sn1—Cl4	90.27 (2)	C10—C11—C12	119.3 (3)
Cl1—Sn1—Cl4	171.72 (2)	C10—C11—H11	120.3
C1—O1—Sn1	118.10 (17)	C12—C11—H11	120.3
C8—O3—H3	110 (2)	C13—C12—C11	118.9 (2)
C6—N1—C2	119.9 (2)	C13—C12—C14	116.9 (2)
C6—N1—Sn1	127.34 (17)	C11—C12—C14	124.3 (3)
C2—N1—Sn1	112.47 (17)	N2—C13—C12	119.2 (2)
C16—N2—C13	124.1 (2)	N2—C13—C8	119.7 (2)
C16—N2—H2	119 (2)	C12—C13—C8	121.1 (2)
C13—N2—H2	117 (2)	C15—C14—C12	121.4 (3)
O2—C1—O1	124.5 (3)	C15—C14—H14	119.3
O2—C1—C2	118.5 (2)	C12—C14—H14	119.3
O1—C1—C2	117.0 (2)	C14—C15—C16	120.3 (3)
N1—C2—C3	121.4 (3)	C14—C15—H15	119.9
N1—C2—C1	115.6 (2)	C16—C15—H15	119.9
C3—C2—C1	123.0 (2)	N2—C16—C15	118.1 (3)
C2—C3—C4	118.7 (3)	N2—C16—C17	119.5 (2)
C2—C3—H3A	120.6	C15—C16—C17	122.4 (2)
C4—C3—H3A	120.6	C16—C17—H17A	109.5
C3—C4—C5	119.5 (3)	C16—C17—H17B	109.5
C3—C4—H4	120.2	H17A—C17—H17B	109.5
C5—C4—H4	120.2	C16—C17—H17C	109.5
C6—C5—C4	118.7 (3)	H17A—C17—H17C	109.5
C6—C5—H5	120.6	H17B—C17—H17C	109.5
			
N1—Sn1—O1—C1	−5.35 (18)	C3—C4—C5—C6	−2.3 (4)
Cl2—Sn1—O1—C1	173.35 (18)	C2—N1—C6—C5	0.8 (4)
Cl3—Sn1—O1—C1	−16.0 (4)	Sn1—N1—C6—C5	−172.80 (19)
Cl1—Sn1—O1—C1	79.65 (18)	C4—C5—C6—N1	1.4 (4)
Cl4—Sn1—O1—C1	−93.15 (18)	O3—C8—C9—C10	180.0 (3)
O1—Sn1—N1—C6	−178.3 (2)	C13—C8—C9—C10	1.6 (4)
Cl2—Sn1—N1—C6	175.77 (18)	C8—C9—C10—C11	1.8 (5)
Cl3—Sn1—N1—C6	−0.3 (2)	C9—C10—C11—C12	−2.7 (4)
Cl1—Sn1—N1—C6	91.8 (2)	C10—C11—C12—C13	0.2 (4)
Cl4—Sn1—N1—C6	−90.5 (2)	C10—C11—C12—C14	−178.6 (3)
O1—Sn1—N1—C2	7.74 (17)	C16—N2—C13—C12	0.4 (4)
Cl2—Sn1—N1—C2	1.8 (4)	C16—N2—C13—C8	−180.0 (3)
Cl3—Sn1—N1—C2	−174.30 (17)	C11—C12—C13—N2	−177.1 (3)
Cl1—Sn1—N1—C2	−82.18 (17)	C14—C12—C13—N2	1.7 (4)
Cl4—Sn1—N1—C2	95.58 (17)	C11—C12—C13—C8	3.3 (4)
Sn1—O1—C1—O2	−179.3 (2)	C14—C12—C13—C8	−177.9 (2)
Sn1—O1—C1—C2	2.3 (3)	O3—C8—C13—N2	−2.3 (4)
C6—N1—C2—C3	−2.0 (4)	C9—C8—C13—N2	176.3 (3)
Sn1—N1—C2—C3	172.4 (2)	O3—C8—C13—C12	177.3 (2)
C6—N1—C2—C1	176.5 (2)	C9—C8—C13—C12	−4.1 (4)
Sn1—N1—C2—C1	−9.1 (3)	C13—C12—C14—C15	−2.4 (4)
O2—C1—C2—N1	−173.5 (2)	C11—C12—C14—C15	176.4 (3)
O1—C1—C2—N1	5.0 (4)	C12—C14—C15—C16	0.9 (4)
O2—C1—C2—C3	5.0 (4)	C13—N2—C16—C15	−1.9 (4)
O1—C1—C2—C3	−176.5 (2)	C13—N2—C16—C17	176.6 (3)
N1—C2—C3—C4	1.1 (4)	C14—C15—C16—N2	1.2 (4)
C1—C2—C3—C4	−177.3 (3)	C14—C15—C16—C17	−177.2 (3)
C2—C3—C4—C5	1.1 (4)		

### Hydrogen-bond geometry (Å, °)

**Table:**

*D*—H···*A*	*D*—H	H···*A*	*D*···*A*	*D*—H···*A*
O3—H3···O2	0.84 (3)	1.86 (1)	2.686 (3)	168 (3)
